# Parenting Styles and Home Obesogenic Environments

**DOI:** 10.3390/ijerph9041411

**Published:** 2012-04-16

**Authors:** Rachel Johnson, Greg Welk, Pedro F. Saint-Maurice, Michelle Ihmels

**Affiliations:** Nutrition and Wellness Research Center, Department of Kinesiology, Iowa State University, Suite 6100, 2325 North Loop Drive, Ames, IA 50011, USA; Email: rachelcdph@gmail.com (R.J.); pedrosm@iastate.edu (P.F.S.-M.); mihmels@iastate.edu (M.I.)

**Keywords:** children, parenting style, home environment, obesity

## Abstract

Parenting behaviors are known to have a major impact on childhood obesity but it has proven difficult to isolate the specific mechanism of influence. The present study uses Baumrind’s parenting typologies (authoritative, authoritarian, and permissive) to examine associations between parenting styles and parenting practices associated with childhood obesity. Data were collected from a diverse sample of children (n = 182, ages 7–10) in an urban school district in the United States. Parenting behaviors were assessed with the Parenting Styles and Dimension Questionnaire (PSDQ), a 58-item survey that categorizes parenting practices into three styles: authoritative, authoritarian, and permissive. Parent perceptions of the home obesogenic environment were assessed with the Family Nutrition and Physical Activity (FNPA) instrument, a simple 10 item instrument that has been shown in previous research to predict risk for overweight. Cluster analyses were used to identify patterns in the PSDQ data and these clusters were related to FNPA scores and measured BMI values in children (using ANCOVA analyses that controlled for parent income and education) to examine the impact of parenting styles on risk of overweight/obesity. The FNPA score was positively (and significantly) associated with scores on the authoritative parenting scale (r = 0.29) but negatively (and significantly) associated with scores on the authoritarian scale (r = −0.22) and permissive scale (r = −0.20). Permissive parenting was significantly associated with BMIz score but this is the only dimension that exhibited a relationship with BMI. A three-cluster solution explained 40.5% of the total variance and clusters were distinguishable by low and high z-scores on different PSDQ sub-dimensions. A cluster characterized as Permissive/Authoritarian (Cluster 2) had significantly lower FNPA scores (more obesogenic) than clusters characterized as Authoritative (Cluster 1) or Authoritarian/Authoritative (Cluster 3) after controlling for family income and parent education. No direct effects of cluster were evident on the BMI outcomes but the patterns were consistent with the FNPA outcomes. The results suggest that a permissive parenting style is associated with more obesogenic environments while an authoritative parenting style is associated with less obesogenic environments.

## 1. Introduction

An ‘*obesogenic*’ environment that contributes to overeating and inactivity has been implicated as a contributing factor in the obesity epidemic. Environmental factors influence behavior in all segments of the population but the issues are unique with children because parents or caregivers dictate the physical and social environments that youth have access to. Parents directly influence a child’s access to healthy or unhealthy foods and enable or inhibit physical activity and sedentary behaviors at home [1,2]. Obesity-related research has examined parenting influence through a variety of mechanisms including practices or policies [3,4,5,6], role modeling [3,7,8,9], and environment/access [10,11,12]. A gap in the literature is a better understanding of the factors that influence or determine these underlying parenting behaviors and practices.

Baumrind’s original characterization of unique parenting typologies [13] provides a useful model for examining parenting styles and practices related to obesogenic environments. This model identifies three distinct parenting styles (Authoritarian, Authoritative, and Permissive). The Authoritarian dimension is characterized by clear parental authority, unquestioning obedience and punitive strategies; the Authoritative dimension is characterized by warmth and involvement, reasoning/induction, and democratic participation; the Permissive dimension is characterized by tolerance, general acceptance of child’s decisions and tendencies to ignore misbehavior. A fourth typology of Uninvolved parenting, characterized by permissiveness with little or no warmth, has also been proposed in an alternative depiction of parenting styles [14]. This adaptation enables parenting style to be characterized into dimensions of demandingness (extent of boundaries/limits) and responsiveness (extent of involvement/warmth).

Studies have used concepts of parenting style to explain a variety of child outcomes including lifestyle factors such as healthy eating [15,16,17,18], physical activity [19,20] and television watching [21,22]. In general, authoritative parenting is thought of as a more positive parenting style but findings with lifestyle behaviors are mixed. Studies, for example, have reported positive associations between permissive parenting and physical activity [19,20,23] but negative associations with television viewing habits [22]. While findings are not completely consistent, the general consensus is that authoritative parenting styles are associated with healthier lifestyles and environments [23,24]. A number of studies have examined specific associations with child weight status but these results have also been somewhat equivocal. Blissett and Haycraft [25] found no correlation between parenting styles and child BMI but Zeller *et al*. [26] demonstrated that permissive parenting and child’s temperament increased the odds of the child being overweight. Increased risks for overweight have been reported for both authoritarian parenting [24,27] and permissive parenting [28]. In contrast, authoritative parenting has been shown to potentially decrease risk [23]. The somewhat equivocal findings in this line of research have led some investigators to conclude that associations between parenting style, parenting practices and child weight status may interact with child or parent characteristics in more complex ways [6,29].

Research on parenting style is complicated by a number of factors. One primary challenge is that it is difficult to distinguish among the different parenting styles since parents can exhibit elements of each parenting dimension. Researchers have supported this notion that parents typically cannot be characterized into a single parenting style [30]. Another challenge is that it is difficult to isolate the impact of parenting style on lifestyle factors since behaviors are influenced by a complex web of social and environmental factors. Parenting styles have been shown to differ due to ethnicity [31] but low-income families tend to have more authoritarian typologies regardless of ethnicity [32,33]. Prevalence of overweight tends to be higher in low income, minority families [10,34,35]. Thus, the somewhat discrepant findings in the literature on parenting style may be due to the lack of attention given to socio-economic status and ethnicity.

The present study is designed to address some of these limitations. The specific purpose was to examine associations between parenting styles and indicators of an obesogenic home environment while controlling for variability due to SES and ethnicity. The study uses a validated behavioral instrument called the Family Nutrition and Physical Activity (FNPA) screening tool [36] to evaluate the home obesogenic environment. Most studies to date have examined diet and activity separately but the FNPA tool provides a more comprehensive evaluation of the home obesogenic environment. The study takes the complexity of parenting styles into account by employing cluster analyses that takes into consideration the unique patterns of parenting styles in the population. Lastly, the study statistically controls for potential confounding variables (SES and ethnicity) to enable the impact of parenting styles to be directly examined. The literature summarized above suggests that authoritative parenting styles tend to produce more positive home environments while authoritarian and permissive parenting styles are associated with less favorable home environments. Therefore we hypothesize that high scores on authoritative and/or low scores on authoritarian or permissive scales would be expected to be associated with better home environments (higher FNPA scores and lower prevalence of overweight). Opposite patterns for these variables would be expected to have the opposite effect (lower FNPA scores and higher prevalence of overweight).

## 2. Experimental Section

### 2.1. Sample Population and Participants

Data were collected from two elementary schools in a large urban school district in the United States. One school (School 1) was in a more diverse, low SES neighborhood in which 80.1% qualified for free and reduced lunch while the other school (School 2) was located in a less diverse, high socio-economic environment (SES) neighborhood in which 30.4% qualified for free and reduced lunch. The ethnic breakdown of School 1 was 58.8% Caucasian, 16.2% Latino, 8.8% African-American, 8.8% Asian or Pacific Islander, and 7.4% Other or Multi-racial while at School 2 was 89.3% Caucasian, 1.9% Latino, 3.9% African-American, 1.0% Asian or Pacific Islander, and 3.9% Other or Multi-racial.

Both schools participated in a BMI screening program as part of the normal district programming. Height and weight data were collected from the available students in the two schools (average age of the elementary students was 8.6 ± 1.7 (5–11 years). BMI was computed from this data with a mean BMI for students of 19 ± 4 kg/m^2^ and this corresponded to an average BMI percentile of 68.3% ± 28.3. Based on the accepted Center for Disease Control (CDC) definitions, approximately 61% of participants were normal weight, 18% were overweight (85th–95th percentile) and 21% were classified as obese (>95th percentile). The distributions for males (48%, 22%, 26%) were slightly different than females (64%, 15%, 17%). Parents were recruited from each school to participate in a supplemental survey component of the project and permission was requested to link their survey data to the child’s BMI data.

### 2.2. Measures

The Parenting Styles and Dimensions Questionnaire (PSDQ) is designed to characterize parenting styles of preschool and school-age children [37]. The tool was designed to assess Baumrind’s three main parenting typologies (authoritative, authoritarian, and permissive) [13]. The instrument is an adapted version of the original Parenting Practices Questionnaire, which includes 58 questions scored on a 1–5 Likert scale. The items are clustered into different stylistic dimensions which are then aggregated to create separate scores for each of the three typologies. Reliability of the individual PSDQ scales ranged from 0.91–0.75 [37]. Questions such as “*I ignore our child’s misbehaviors*” help to determine parents’ permissiveness while other questions such as “*I demand for our child to do things*” help to determine parents’ authoritarian styles (four questions were removed from the original questionnaire to avoid reporting types of corporal punishment). There are four dimensions used to characterize the authoritative typology (warmth and involvement, reasoning/induction, democratic participation, good natured/easy going), four used for authoritarian (verbal hostility, non-reasoning/punitive strategies directiveness) and three for permissive (lack of follow through, ignoring misbehavior, self-confidence). The number of items in each stylistic dimension varied so mean scores were first computed for each dimension. The total composite score for each parenting typology was then determined by computing an average of each of the associated stylistic dimensions. This method weights each stylistic dimension equally rather than basing the overall typology on the mean of all associated items. We used cluster analyses to determine primary parenting styles since there is no specific classification scheme available to determine a predominant style (see cluster procedures below).

The Family Nutrition and Physical Activity (FNPA) assessment was used to collect detailed information on home environments and behaviors that may predispose youth to become overweight or obese. Based on findings from a comprehensive evidence analyses, the FNPA specifically assesses ten risk factors (constructs) found to be consistently associated with overweight/obesity in children: (1) breakfast patterns; (2) family eating; (3) food choices; (4) beverage choices; (5) parental restriction and reward; (6) TV/video game/computer screen time; (7) TV usage; (8) family activity; (9) child physical activity; and (10) family bedtime routine. The items on the FNPA tool have been shown to load on a single factor and to have good internal reliability (alpha = 0.72) [36]. The predictive validity of the FNPA tool was also supported in a longitudinal study that demonstrated the utility of the FNPA for detecting potential risk of child overweight [38]. The original FNPA tool used Likert type scales but a modified version using behaviorally anchored rating (BAR) scales was used to facilitate data collection in the present study. Responses on the 10 items are scored on a 3 point scale (1 is more obesogenic and 3 is less obesogenic) so the total scores ranged from 10 to 30. A Spanish version of the FNPA was created for the sizable proportion of parents whose first language was Spanish. The BAR format reduces the likelihood of socially desirable responses by allowing parents to select the environment that most closely fits their family. Other instruments have been developed to measure home environments [39,40,41] but the FNPA is unique in capturing diverse aspects of the home environment that may predispose youth to become overweight.

### 2.3. Procedures

The sensitive nature of the study necessitated the use of procedures to ensure confidentiality of the participants. Specifically, the study required merging children’s measured BMI data with parent’s self-report data in a confidential manner. The schools conducted BMI screening as part of normal physical education assessments but trained members of the research team provided equipment and conducted the measurements for the study. A Tanita BF-681W (Tokyo, Japan) scale was used to measure weight and a Seca Road Rod stadiometer (Hanover, MD, USA) was used to measure height. The height and weight data were recorded on a form along with student ID to facilitate tracking and computation of BMI. After students completed the BMI testing they were provided with a survey packet to take home to their parents that contained the FNPA and PSDQ forms along with the associated informed consent form. The student ID numbers from class lists were pre-printed on the FNPA and PSDQ surveys to enable these to be completed by the parent and returned in a confidential manner. A second set of packets were distributed to parents two weeks after the first distribution, for parents who had yet to complete and return the surveys. The study protocol was approved by the Iowa State University Institutional Review Board as well as the participating school district.

### 2.4. Analyses

Descriptive analyses were conducted to examine the differences between the schools in terms of demographics, child BMI, parenting style and home environment. We conducted independent t-tests (for continuous variables) and Pearson chi-square tests (for categorical variables) to assess significant differences on demographic information between schools. Correlation analyses (using Pearson product moment correlations) were performed to examine cross sectional associations among the study variables (PSDQ dimensions, FNPA score and child BMI percentiles). The primary statistical analyses involved the use of cluster analyses to investigate the typology of parenting style and subsequent group comparisons to explore how the clusters related to FNPA and BMI percentile scores. In order to increase statistical power, data from two schools were combined before running the cluster analyses.

Cluster Assumptions: standardization is highly recommended in cluster analyses to enable different variable scales to be directly compared. Standardization also ensures that all variables have the same impact on similarities avoiding the influence of differences in scales. Based on an initial sample of 182 clusters, traditional statistical procedures were used to screen the data for normalization, outliers (z ≥ 3.5), and implicit weighting (correlations between PSDQ dimensions). A correlation value of ≥0.40 was used as an indicator of highly correlated variables which generated an implicit weighting of the concepts that the study was measuring. Data were then standardized and high z-scores were defined as z-scores above 0.5 standard deviations (SD) while low scores were defined as −0.5 SD. A z-score between −0.5 to 0.5 SD would then represent an average score on any of the variables considered.

Cluster Determination: clusters were determined using similarity coefficients, which reflect an overall indicator of dissimilarity. This method (commonly used in both social and natural sciences) was preferred in order to increase the differences between clusters. This method merges the two most similar cases and repeats the process until there is only one cluster solution. The Ward´s method for establishing the cluster was based on case linkage and used to optimize the minimum variance within clusters. Determination of the final number of clusters was based on commonly used cluster analysis techniques—including visual inspection of the dendogram, evaluation of relevant indices that reveal loss of information when clusters/cases are merged (e.g., fusion coefficient), value of the pseudo F statistic, and interpretation of expected and actual R-square.

Evaluation of Cluster Differences: these analyses were conducted on standardized scores (z-scores) for all the variables of interest. We used the cluster solution obtained from the previous analyses to describe the association between clusters, and standardized FNPA item scores. This allowed for a more comprehensive interpretation on possible FNPA score differences between clusters. We then looked at cluster differences on total FNPA standardized scores and BMI percentile z-scores. The standardization of BMI percentile scores allowed for relative comparisons within our sample. These associations were tested using two separate ANCOVAS for each of the outcomes using income and education as covariates. The key assumptions required by this statistical method were assessed and included—data normality (visually determined), homoscedasticity (based on scatter plots and Leven´s test), and homogeneity of regression (conducted an ANOVA to test slopes using interaction terms between independent variable and each covariate). We were particularly concerned with meeting the homogeneity of regression assumption. Since our initial ANCOVA model did not meet this requirement we decided to adjust our model to account for unequal slopes. Therefore, multiple comparison tests were based on Least Square Means tests (using Tukey) adjusting for covariates. Adjusted covariate values were set at half range score or their respective median (income was set at a value of 2—$25,000–50,000 and education was set at a value of 3—“attended some college”) in order to better represent average educated and average income population scores. Thus this step is critical otherwise differences in FNPA or BMI percentile scores will greatly depend on the covariate value. This approach is recommended when homogeneity of regression condition is rejected, so inferences relate to the effect of clusters holding income and education at a reasonable value [42]. Post assumptions testing were done to further assess the performance of the model created. Those included—normality of residuals (inspected visually), relation between residuals, and predicted values and also covariates (inspected with an ANOVA and based on visual distribution using scatter plots). Effect size for the cluster effect on FNPA and BMI percentile was computed using omega squared and these were interpreted using Cohen guidelines [43]. Statistical significance was identified based on a *p* < 0.05. Data was processed and analyzed using SAS v9.2 software (SAS Institute Inc., Cary, NC, USA).

## 3. Results

Complete data on family demographics (e.g., ethnicity, income, education), selected cluster variables (*i.e.*, PSDQ) and the two outcomes of interest (FNPA and BMI percentile) were available for 171 out of the total sample of 182 participants (School 1 = 68 and School 2 = 103). The demographic profiles and descriptive statistics on the samples are provided in Table 1. Students at School 1 and School 2 had a similar age (t_169_ = −1.50, *p* = 0.135) and weight (t_169_ = −1.96, *p* = 0.051) but differed in height (t_169_ = −3.86, *p* < 0.001). The ethnic distributions were significantly different between schools (*x*^2^ = 26.92, *p* < 0.001). The overall percentage of Caucasians was slightly over-represented and the minorities slightly under-represented but the distributions mirrored the overall demographics in each school. Parent level of education and average income differed between schools (*x*^2^ = 63.27, *p* < 0.001 and *x*^2^ = 70.15, *p* < 0.001, respectively). Most of the parents at School 2 (79.7%) reported earning $50,000 or more, the majority of parents at School 1 reported earning less than $50,000 (85.3%). While there is some selection bias in the sample (due to the voluntary completion of the survey) the use of two schools provided a more representative and diverse sample for the analyses. The data from the two schools were combined for the remaining analyses.

**Table 1:** Student and Parent demographics by school.

	School 1 (n = 68)	School 2 (n = 103)	Mean Diff.		*p*−value
				T	
Age (years) *	8.6 (1.7)	8.9 (1.3)	−0.3	−1.50	0.135
Height (cm) *	130.4 (11.7)	136.6 (9.4)	−6.2	−3.86	<0.001
Weight (kg) *	32.2 (11.7)	35.6 (10.6)	−3.4	−1.96	0.051
				*x*^2^	
Race (%)				26.92	<0.001
Asian or Pacific Islander	8.8	1.0	7.8		
African-American	8.8	3.9	4.9		
Caucasian	58.8	89.3	−30.5		
Latino	16.2	1.9	14.3		
Multi-racial	7.4	3.9	3.5		
Education (%)				63.27	<0.001
Some HS	14.7	1.0	13.7		
HS Grad	23.5	4.9	18.6		
Some College	36.8	14.6	22.2		
College Grad	25.0	51.5	−26.5		
Graduate Degree	0	28.2	−28.2		
Income (%)				70.15	<0.001
<$25,000	45.6	8.7	36.9		
$25–50,000	38.2	11.7	26.5		
$50–75,000	7.4	10.7	−3.3		
>$75,000	8.8	68.9	−60.1		

* Values are Mean (SD).

Correlations among study variables were computed to assist in interpretation of the results and to facilitate comparisons with other studies. All the reported correlations were statistically significant (*p* < 0.05). The correlation between FNPA and child BMI percentile z-scores was negative and statistically significant (r = −0.23), and similar in magnitude to the correlations reported in the original FNPA paper [36]. Further, FNPA score was positively associated with score on the authoritative parenting scale (r = 0.29) but negatively associated with scores on the authoritarian scale (r = −0.22) and permissive scale (r = −0.20). Permissive parenting was positively associated with BMI percentile z-scores (r = 0.16) but this was the only dimension that exhibited a relationship with this indicator. Correlational analysis also indicated an association between some of the PSDQ dimensions. Permissive parenting scores were positively associated with authoritarian scores (r = 0.54), while authoritarian scores were inversely related to authoritative scores (r = −0.25).

**Figure 1:**
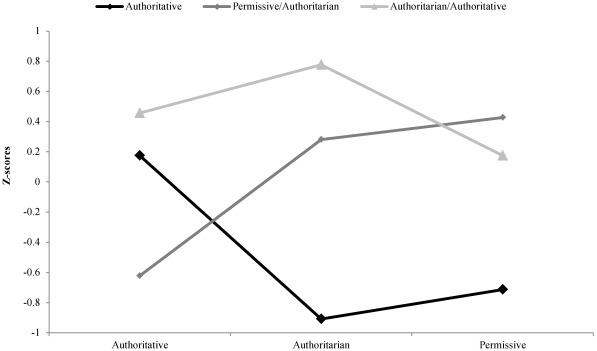
Average Z-scores for the three-cluster parenting style.

Cluster analyses were conducted to classify parents into the different parenting styles since there are no published procedures for interpreting the PSDQ data. The PSDQ data were normally distributed and met the assumptions required for cluster analyses. Preliminary analyses of the dendogram, fusion coefficient “elbows”, pseudo-F statistic and r^2^ distributions suggested that the optimum number of clusters was either three or four and therefore both solutions were examined in more detail to determine the most optimal fit. The four-cluster solution was associated with higher proportion of explained variance; however the fourth factor was not clearly interpretable (*i.e.*, there was no clear pattern among the typologies). Our limited sample size did not allow a detailed exploratory analysis to determine the appropriate number of clusters but the three cluster solution provided a good fit and yielded clearly interpretable factors. The three-cluster solution explained 40.5% of the total variance and clusters were distinguishable by low and high z-scores on different PSDQ sub-dimensions. Cluster 1 participants (n = 63) were characterized by slightly positive values on Authoritative (0.17 ± 1.10), but low scores on Permissive (−0.71 ± 0.59) and Authoritarian (−0.91 ± 0.52). Participants in Cluster 2 (n = 57) were characterized by higher than average scores on Permissive (0.43 ± 0.62), Authoritarian (0.28 ± 0.64) but low values on Authoritative (−0.62 ± 0.71). Cluster 3 participants (n = 51) had average scores on Permissive (0.17 ± 0.87) and Authoritative (0.46 ± 0.70), but high scores on the Authoritarian scale (0.78 ± 0.78). Based on these scores we defined Cluster 1 as Authoritative, Cluster 2 as Permissive/Authoritarian, and Cluster 3 as Authoritarian/Authoritative (Figure 1).

**Figure 2:**
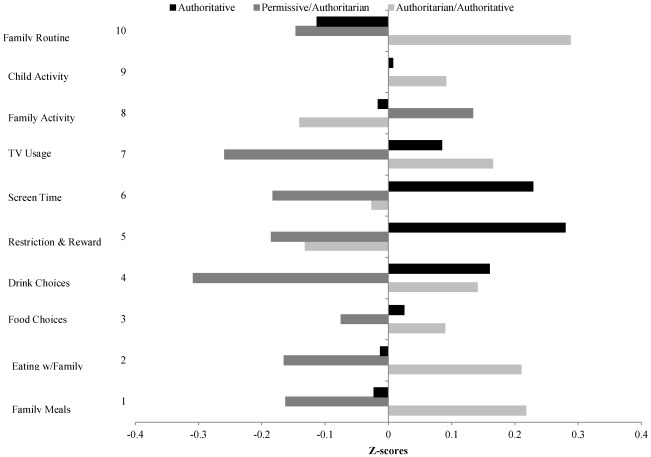
Cluster average z-scores in individual FNPA items.

The clusters were related to scores on individual FNPA items to examine the impact of parenting styles on specific home environments (see Figure 2). Parents classified into Cluster 1 (Authoritative) had a positive z-score on item 5 (mean = 0.28 ± 0.89) and item 6 (mean = 0.23 ± 0.77). These two items assessed monitoring of food and TV time so the higher scores on this cluster suggest that parents in this cluster were more likely to monitor consumption of snacks and limit time spent watching a screen. Parents classified into Cluster 2 (Permissive/Authoritarian) had a negative z-score on item 4 (mean = −0.31 ± 1.12) and 7 (mean = −0.26 ± 1.10). These items assessed drink choices and T.V. usage so the negative scores suggest less parental control on these aspects of the home environment. Parents classified into Cluster 3 (Authoritarian/Authoritative) had a distinguishable positive score on item 10 (mean 0.29 ± 1.07). This item assessed family routine so the high value here suggests that this item is the most distinguished classification into this cluster. Scores on the remaining items ranged between −0.19 and 0.21. The clusters were related to overall FNPA and BMI percentile scores to examine the overall impact of parenting styles on risk for overweight. Separate ANCOVA analyses were used to control for effects of income and education on both outcomes.

The FNPA scores were normally distributed so no data transformations were performed. Homocedasticity was confirmed by random patterns in FNPA variability scores across all variables in the model and non-significant p-values from the Levene test (*p* > 0.05). The ANOVA (univariate tests for interaction term) indicated that the interaction between cluster and each covariate was significant when “testing” for homogeneity of regression (*p* < 0.05). Therefore, it was decided to keep both covariates and to account for unequal slopes in the ANCOVA model. The adjusted FNPA z-scores (with income and education set at average scores) differed between clusters (F_2,162_ = 4.68, *p* = 0.011). The FNPA z-score for Cluster 2 (z_Permissive/Authoritarian_ = −0.54 ± 0.14) was significantly lower than the value for Cluster 1 (z_Authoritative_ = −0.01 ± 0.12) and Cluster 3 (z_Authoritarian/Authoritative_ = −0.01 ± 0.15), and the overall effect of cluster on FNPA scores was small (ω^2^ = 0.03). Further inspection of the accuracy of our model revealed normally distributed residuals, with no significant differences or systematic patterns in residual scores among income (*p* = 0.719) and education groups (*p* = 0.229).

**Figure 3:**
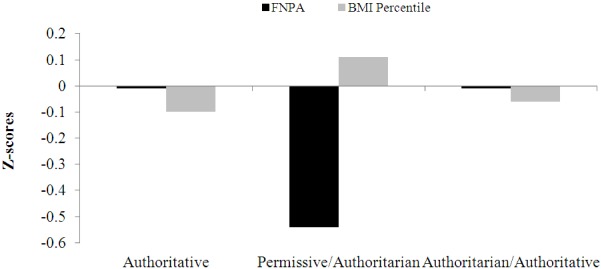
Clusters average z-scores on FNPA and BMI percentile.

The BMI percentile z-scores were slightly skewed to the right however they had equal variance across all variables in the model, supported by non-significant p-values from the Levene test (*p* > 0.05). The ANOVA revealed no significant interactions between cluster and each covariate (homogeneity of regression) (*p* > 0.05). This indicated that variability in BMI percentile z-scores was constant across clusters at different levels of each covariate. Nevertheless, despite homogeneity of regression, it was decided to employ the same unequal slopes model so the two models (FNPA and BMI) could be directly compared. The adjusted BMI percentile z-scores (with income and education set at average scores) did not differ between clusters (F_2,162_ = 0.50, *p *= 0.605). Despite overall non-significant differences between clusters, Cluster 2 (Permissive/Authoritarian) had the highest BMI percentile z-score (0.11 ± 0.17). BMI percentile z-scores in Cluster 1 (Authoritative) and Cluster 3 (Authoritarian/Authoritative) were similar (z_Authoritative_ = −0.10 ± 0.14 and z_Authoritarian/Authoritative_ = 0.06 ± 0.17). See Table 2 for exact scores and Figure 3 for visual inspection of the cluster pattern among schools in FNPA and BMI percentile z-scores. There was no effect of cluster typology on BMI percentile z-scores (ω^2^ = 0.00). Residuals were normally distributed, with no significant differences or systematic patterns in residual scores among income (*p* = 0.792) and education groups (*p* = 0.941).

**Table 2:** Cluster by scores in parenting style, FNPA and BMI percentile scores.

	Authoritative	Permissive/Authoritarian	Authoritarian/Authoritative
	(n = 63)	(n = 57)	(n = 51)
Authoritative ^1^	0.65 ± 0.74	−0.27 ± 0.43	0.78 ± 0.56
Authoritarian ^1^	−0.99 ± 0.48	0.09 ± 0.61	0.40 ± 0.60
Permissive ^1^	−0.56 ± 0.53	0.37 ± 0.56	0.10 ± 0.80
FNPA ^2^	−0.01 ± 0.12	−0.54 ± 0.14	−0.01 ± 0.15
BMI percentile ^2^	−0.10 ± 0.14	0.11 ± 0.17	−0.06 ± 0.17

^1^ Mean ± Standard Deviation; ^2^ Adjusted Mean ± Standard Error.

## 4. Discussion

Parents exert perhaps the strongest and most direct influence on a child’s potential risk for overweight and obesity [1,2]. Considerable attention has been placed on the importance of genetic factors but emphasis in this paper is on the social and behavioral factors that may explain parenting practices within the home environment. Parents serve as gatekeepers by restricting or enabling access to foods and physical activity opportunities [10,11,12]. They also shape and influence behavior by establishing rules and standards for family meals, sleep schedules and other lifestyle behaviors [11,12,20,21,22]. Finally, parents shape and influence behavior through modeling and support of healthy behaviors [3,7,8,9]. Research shows that child characteristics may interact with parenting styles and practices to influence child behaviors and outcomes [4,29]. The numerous factors and nested layers of influence have made it very difficult to fully understand parental influences. The incomplete understanding of mechanisms underlying parenting practices, in turn, may explain the limited utility of most family-based interventions.

In the present study we evaluated whether Baumrind’s parenting typologies could explain differences in home obesogenic environments. Previous research has shown some utility for this approach but it has proven difficult to use and interpret since parents can have characteristics of several parenting styles (*i.e.*, parents can be classified as more or less authoritative or more or less permissive *etc*.). An advantage of the present study is that we employed cluster analyses to identify naturally occurring clusters or patterns in the data. This allowed the value for each typology to be interpretable relative to others in the sample rather than using arbitrary criteria.

The cluster analyses resulted in three distinct parenting profiles. Cluster 1 was characterized as predominantly Authoritative primarily because of the extremely low z-scores for Permissive and Authoritarian. Cluster 2 was characterized as Permissive/Authoritarian due to above average z-scores on these two dimensions and low scores on Authoritative. Cluster 3 was characterized as Authoritarian/Authoritative due to the high scores on these dimensions and low scores on Permissive. As expected, the resulting clusters demonstrate some hybridization among the three parenting typologies. Cluster 1 and Cluster 3 both had above average values for Authoritative behaviors but the difference was on the degree of Authoritarian behaviors. Specifically, Cluster 1 had below average (low) values on Authoritarian dimension while Cluster 3 had above average (high) values on the Authoritarian dimension. The Authoritative components of warmth, involvement and participation were apparently evident in both clusters but the groups were distinguished by the degree (and nature) of parenting authority. Cluster 2 was characterized by tendencies for both Authoritarian and Permissive parenting, but these parents apparently did not report characteristics of warmth, involvement and participation as the scores on the Authoritative dimension were low. The blending of styles is consistent with the concept of “gray areas” within Baumrind’s typologies [30]. The various stylistic dimensions and associated parenting typologies operate on continuums with parents occasionally exhibiting characteristics of multiple styles. 

The results confirmed that parenting styles had an impact on the FNPA scores as significant differences were evident in FNPA scores across the various clusters. Parents characterized in Cluster 2 (Permissive/Authoritarian) were found to have significantly lower FNPA scores (less healthy home environments) than parents in Cluster 1 or Cluster 3. These results are consistent with other studies [27,44,45] that have reported that permissive parenting was associated with behaviors that are related to weight gain in children. The findings of the study are also consistent with findings showing a tendency for more favorable child outcomes from more authoritative parents [23,24]. 

The present study provided a more robust evaluation of these patterns as we statistically controlled for education and income in the analyses. Research has shown that family income, education, and neighborhood SES are associated with a child’s obesity risk. More specifically, studies have shown that children raised in lower income families (or from lower SES neighborhoods) have a higher risk for overweight [34,46,47]. This pattern was also evident in the present sample as we observed significantly higher prevalence of overweight in the low income, high minority school. However, by controlling for income and education we are able to conclude that the differences in FNPA scores are attributable to differences in parenting style and not due directly to income or education. 

While parenting style was found to be associated with FNPA score none of the parenting styles were significant predictors of child BMI. Results from others studies reported similar findings [19,25]. This is understandable since a number of other variables can influence a child’s BMI. Some families, for example, may have good dietary and activity habits/environments but be genetically predisposed to overweight/obesity. The genetic influence on BMI may have a stronger (over-riding) impact on BMI than the home environmental factors evaluated in this study—but this possibility cannot be evaluated. The clear pattern shown for FNPA outcomes suggests that parenting styles may be an important indicator or precursor of potential risk. A previous longitudinal study by our team demonstrated that FNPA scores were associated with change in BMI over a one year follow up [38]. Therefore, it is possible that the parenting styles may operate in subtle ways over time to influence parenting behaviors and children’s future risk for overweight and obesity. 

## 5. Conclusions

The results of the study demonstrate the utility of parenting style and the PSDQ tool for evaluating parenting influence on shaping home obesogenic environments. Consistent with our hypotheses, we found that a more permissive parenting style was associated with a more obesogenic environment while a more authoritative parenting style was associated with a less obesogenic environment. The differential relationships observed for some of the specific FNPA items shows that parenting styles may be associated with unique tendencies to monitor, limit or promote specific behaviors in children. These insights may prove useful in determining appropriate targets for interventions. This study provides new insights into the relationships between parenting style and home obesogenic environments but some specific limitations should be considered when interpreting the results. One key limitation to this study is the relatively low response rate. A low response rate could limit the generalizability of the results but the use of two schools helped ensure that there was a reasonably diverse and more representative sample. Another limitation is the inability to control for parent BMI. A longitudinal study demonstrated that increases in BMI in overweight parents and their overweight children were associated with corresponding increases in snacking and television viewing time [48]. Another study [21] showed that parental rules limiting children’s TV viewing time were more common in children whose parents were not overweight. It is possible that parent BMI is associated with (or caused by) the same underlying home environment, but it is also possible that weight status may influence parenting style and practices either directly or indirectly. The cross sectional nature of the present study does not enable us to examine these issues. A third limitation is the inability to look at alternative conceptions of parenting style. The addition of an “*Uninvolved/Passive*” parenting dimension could have led to more definitive distinctions among parents and a different relationship among clusters. A paper by Hennessy *et al*. [19] reported that Uninvolved parenting was the most common style so this deserves further attention. Lastly, it is possible that the parenting styles work through other mechanisms. The present study provides some evidence that parenting styles are related to home environments (as assessed with the FNPA) but it is possible that other unmeasured factors could influence lifestyles and risk for overweight. The FNPA tool was developed primarily as a screening tool and may not provide sufficient depth to fully characterize home environments. Additional studies using longitudinal designs are needed to examine the impact of parenting styles and home environments over time.
